# (*E*)-4-(4-Fluoro­styr­yl)benzoic acid

**DOI:** 10.1107/S1600536808012920

**Published:** 2008-05-07

**Authors:** Saba Nazir, Khushi Muhammad, M. Khawar Rauf, Masahiro Ebihara, Shahid Hameed

**Affiliations:** a Department of Chemistry, Quaid-i-Azam University, Islamabad 45320, Pakistan; b Department of Chemistry, Faculty of Engineering, Gifu University, Yanagido, Gifu 501-1193, Japan

## Abstract

The title compound, C_15_H_11_FO_2_, is an important inter­mediate in the synthesis of side-chain ligands for polymeric liquid crystals. The vinyl group is almost coplanar with both the aromatic rings. The crystal structure is stabilized by inter­molecular O—H⋯O hydrogen bonding.

## Related literature

For related literature, see: Ahmad *et al.* (2003 ▶); Collings & Hird (1997 ▶); Frazee & Foraker (2008 ▶); Hameed & Rama (2004 ▶); Hussain *et al.* (2005 ▶); Nazir *et al.* (2008 ▶); Ribeiro *et al.* (2008 ▶); Wang *et al.* (2008 ▶); Higashi (1999 ▶); Yasuda *et al.* (2000 ▶).
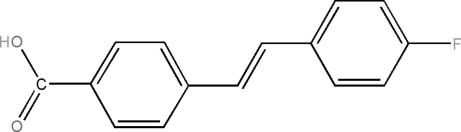


## Experimental

### 

#### Crystal data

C_15_H_11_FO_2_
*M* *_r_* = 242.24
Monoclinic, 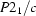

*a* = 6.261 (4) Å
*b* = 23.096 (15) Å
*c* = 8.269 (5) Å
β = 107.072 (8)°
*V* = 1143.1 (13) Å^3^
*Z* = 4
Mo *K*α radiation
μ = 0.10 mm^−1^
*T* = 123 (2) K
0.45 × 0.30 × 0.18 mm

#### Data collection

Rigaku/MSC Mercury CCD diffractometer
Absorption correction: none
9111 measured reflections
2589 independent reflections
2399 reflections with *I* > 2σ(*I*)
*R*_int_ = 0.036

#### Refinement

*R*[*F* ^2^ > 2σ(*F* ^2^)] = 0.065
*wR*(*F* ^2^) = 0.155
*S* = 1.16
2589 reflections
167 parameters
H atoms treated by a mixture of independent and constrained refinement
Δρ_max_ = 0.55 e Å^−3^
Δρ_min_ = −0.28 e Å^−3^

### 

Data collection: *CrystalClear* (MSC/Rigaku, 2001 ▶); cell refinement: *CrystalClear*; data reduction: *TEXSAN* (MSC/Rigaku, 2004 ▶); program(s) used to solve structure: *SIR97* (Altomare *et al.*, 1999 ▶); program(s) used to refine structure: *SHELXL97* (Sheldrick, 2008 ▶); molecular graphics: *ORTEPII* (Johnson, 1976 ▶); software used to prepare material for publication: *SHELXL97* and *TEXSAN*.

## Supplementary Material

Crystal structure: contains datablocks I, global. DOI: 10.1107/S1600536808012920/hg2398sup1.cif

Structure factors: contains datablocks I. DOI: 10.1107/S1600536808012920/hg2398Isup2.hkl

Additional supplementary materials:  crystallographic information; 3D view; checkCIF report

## Figures and Tables

**Table 1:** Hydrogen-bond geometry (Å, °)

*D*—H⋯*A*	*D*—H	H⋯*A*	*D*⋯*A*	*D*—H⋯*A*
O2—H2⋯O1^i^	1.04 (4)	1.57 (4)	2.610 (2)	174 (3)

Symmetry code: (i) 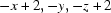.

**Fig. 1.**
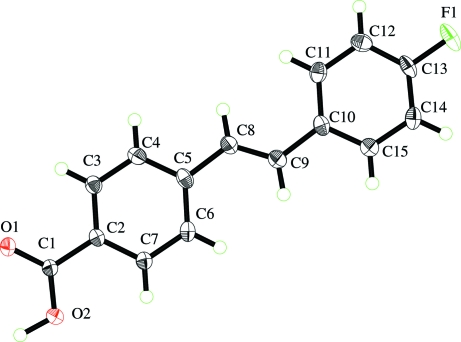
Molecular structure of (I) showing atom-labelling scheme and displacement ellipsoids at the 30% probability level.

**Fig. 2.**
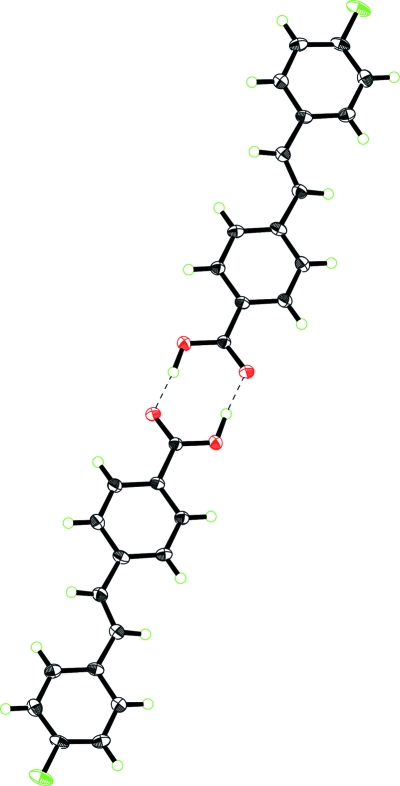
Showing hydrogen bonded molecules through N—H···O.

## supplementary crystallographic information

### Comment

Carboxylic acids constitute an important class of organic compounds and have been used by the medicinal industry as important drugs (Ribeiro *et al.*, 2008; Frazee & Foraker 2008; Hameed & Rama 2004). The carboxylic acids have also found applications as intermediates in the synthesis of an enormous number of organic compounds, in general (Hussain *et al.*, 2005; Ahmad *et al.*, 2003), and in the synthesis of side chain ligands for polymeric liquid crystals, in particular (Wang *et al.*, 2008; Nazir *et al.*, 2008). The liquid crystalline molecules containing substituents at 4-position behave as well ordered calamitic ligands as a side chain group (Collings & Hird, 1997) in side chain polymeric liquid crysrtals (*SCPLCs*). The derivatives of 4-(4-substituted styryl)benzoic acids have found applications as side chain groups in *SCPLCs* (Wang *et al.*, 2008). As a part of a project to synthesize ligands for *SCPLCs*, the title compound, (*E*)-4-(4-fluorostyryl)benzoic acid (I), was synthesized by reacting 4-fluorobenzaldehyde with methyl [4-(methoxycarbonyl)benzyl]triphenylphosphonium bromide (Nazir *et al.*, 2008) followed by hydrolysis. In the present article, the crystal structure of (I) is being reported. Bond lengths and angles are within the normal ranges as given for vinylbenzoic acid (Yasuda *et al.*, 2000). The C(1)—O(1) and C(1)—O(2) bond lengths are 1.252 (2) and 1.291 (2) respectively,clearly indicating the partial double bond character of the carboxylate group. The carboxylic acid group subtends a dihedral angle[13.72 (16)°] with the phenyl ring C(2)/C(3)/C(4)/C(5)/C(6)/C(7).The vinyl group is almost coplanar with both the phenyl rings. The torsion angles between the phenyl rings and vinyl group fulfill the condition of coplanarity[near to 0° or 180 °]. Two molecules related by an inversion center form a dimer *via* two hydrogen bonds composed of two carboxyl groups as shown in Fig. 2.

### Experimental

Methyl 4-(4-fluorostyryl)benzoate 0.8g (0.0031moles) and sodium hydroxide 0.126g (0.0031 moles) were dissolved in a mixture of 10 ml of methanol and 30 ml of water, and the mixture refluxed for 3 hours. The reaction mixture was cooled to room temperature and acidified with 6M HCl. The precipitated solid was filtered and recrystallized from hot ethanol. Yield: 76%, m.p: 240-252°C, R_f_ = 0.22 (n-hexane : ethyl acetate 7 : 3). IR (ν_max_, KBr, cm^-1^): 3300-2500, 1715, 1620, 1600, 1580, 1188, 1119, 965,834. ^1^H-NMR (300 MHz,DMSO-d_6_): δ  7.25 (2H, d, J = 9.0 Hz),7.28 (1H, d, J= 16.2 Hz), 7.42 (1H, d, J = 16.2 Hz), 7.71-7.67 (4H, m),7.95 (2H, d, J = 8.1 Hz), 12.93 (1H, s). ^13^C-NMR (75 MHz, DMSO-d_6_): δ 116.16 (d, J= 23 Hz), 126.90, 127.77, 129.23, 130.08 (d, J = 8 Hz), 131.95, 132.49, 133.71 (d, J = 3 Hz), 141.83, 162.42 (d, J = 246 Hz), 167.54.

### Refinement

The O-bound H atom was refined isotropically. All the other H atoms were placed in idealized positions and treated as riding atoms with C—H distance in the range 0.95–0.99 Å and *U*_iso_(H) = 1.2*U*_eq_(C) or 1.5*U*_eq_(C).

### Crystal data

**Table:**

C_15_H_11_FO_2_	*F*(000) = 504
*M**_r_* = 242.24	*D*_x_ = 1.408 Mg m^−^^3^
Monoclinic, *P*2_1_/*c*	Mo *K*α radiation, λ = 0.71070 Å
Hall symbol: -P 2ybc	Cell parameters from 3252 reflections
*a* = 6.261 (4) Å	θ = 3.1–27.5°
*b* = 23.096 (15) Å	µ = 0.10 mm^−^^1^
*c* = 8.269 (5) Å	*T* = 123 K
β = 107.072 (8)°	Needle, colourless
*V* = 1143.1 (13) Å^3^	0.45 × 0.30 × 0.18 mm
*Z* = 4	

### Data collection

**Table:**

Rigaku/MSC Mercury CCD diffractometer	2399 reflections with *I* > 2σ(*I*)
Radiation source: Rotating anode	*R*_int_ = 0.036
graphite	θ_max_ = 27.5°, θ_min_ = 3.1°
ω scans	*h* = −8→6
9111 measured reflections	*k* = −25→29
2589 independent reflections	*l* = −10→9

### Refinement

**Table:**

Refinement on *F*^2^	Primary atom site location: structure-invariant direct methods
Least-squares matrix: full	Secondary atom site location: difference Fourier map
*R*[*F*^2^ > 2σ(*F*^2^)] = 0.065	Hydrogen site location: inferred from neighbouring sites
*wR*(*F*^2^) = 0.155	H atoms treated by a mixture of independent and constrained refinement
*S* = 1.16	*w* = 1/[σ^2^(*F*_o_^2^) + (0.0616*P*)^2^ + 0.7669*P*] where *P* = (*F*_o_^2^ + 2*F*_c_^2^)/3
2589 reflections	(Δ/σ)_max_ < 0.001
167 parameters	Δρ_max_ = 0.55 e Å^−^^3^
0 restraints	Δρ_min_ = −0.28 e Å^−^^3^

### Special details

**Table:**

Geometry. All e.s.d.'s (except the e.s.d. in the dihedral angle between two l.s. planes) are estimated using the full covariance matrix. The cell e.s.d.'s are taken into account individually in the estimation of e.s.d.'s in distances, angles and torsion angles; correlations between e.s.d.'s in cell parameters are only used when they are defined by crystal symmetry. An approximate (isotropic) treatment of cell e.s.d.'s is used for estimating e.s.d.'s involving l.s. planes.
Refinement. Refinement of *F*^2^ against ALL reflections. The weighted *R*-factor *wR* and goodness of fit *S* are based on *F*^2^, conventional *R*-factors *R* are based on *F*, with *F* set to zero for negative *F*^2^. The threshold expression of *F*^2^ > σ(*F*^2^) is used only for calculating *R*-factors(gt) *etc*. and is not relevant to the choice of reflections for refinement. *R*-factors based on *F*^2^ are statistically about twice as large as those based on *F*, and *R*- factors based on ALL data will be even larger.

### Fractional atomic coordinates and isotropic or equivalent isotropic displacement parameters (Å^2^)

**Table:**

	*x*	*y*	*z*	*U*_iso_*/*U*_eq_	
C1	0.7742 (3)	0.03235 (7)	0.8139 (2)	0.0188 (4)	
O1	0.9748 (2)	0.04569 (6)	0.83715 (16)	0.0268 (3)	
O2	0.7095 (2)	−0.00358 (6)	0.90952 (16)	0.0258 (3)	
H2	0.843 (6)	−0.0202 (16)	1.006 (5)	0.089 (12)*	
C2	0.5982 (3)	0.05820 (7)	0.6720 (2)	0.0188 (4)	
C3	0.6547 (3)	0.10173 (8)	0.5755 (2)	0.0221 (4)	
H3	0.8054	0.1143	0.6002	0.027*	
C4	0.4916 (3)	0.12671 (8)	0.4437 (2)	0.0246 (4)	
H4	0.5313	0.1568	0.3796	0.030*	
C5	0.2699 (3)	0.10851 (8)	0.4031 (2)	0.0231 (4)	
C6	0.2149 (3)	0.06476 (8)	0.5012 (2)	0.0232 (4)	
H6	0.0645	0.0519	0.4756	0.028*	
C7	0.3771 (3)	0.03999 (8)	0.6354 (2)	0.0213 (4)	
H7	0.3374	0.0107	0.7020	0.026*	
C8	0.1067 (3)	0.13727 (8)	0.2605 (2)	0.0250 (4)	
H8	0.1586	0.1694	0.2111	0.030*	
C9	−0.1064 (3)	0.12250 (8)	0.1943 (2)	0.0246 (4)	
H9	−0.1587	0.0904	0.2436	0.030*	
C10	−0.2687 (3)	0.15134 (8)	0.0517 (2)	0.0224 (4)	
C11	−0.2127 (3)	0.19609 (8)	−0.0431 (2)	0.0250 (4)	
H11	−0.0628	0.2094	−0.0148	0.030*	
C12	−0.3734 (3)	0.22120 (8)	−0.1778 (2)	0.0273 (4)	
H12	−0.3353	0.2514	−0.2424	0.033*	
C13	−0.5900 (3)	0.20104 (8)	−0.2150 (2)	0.0262 (4)	
C14	−0.6531 (3)	0.15774 (8)	−0.1256 (2)	0.0257 (4)	
H14	−0.8040	0.1451	−0.1539	0.031*	
C15	−0.4898 (3)	0.13277 (8)	0.0075 (2)	0.0241 (4)	
H15	−0.5300	0.1023	0.0700	0.029*	
F1	−0.7470 (2)	0.22525 (6)	−0.34838 (15)	0.0419 (4)	

### Atomic displacement parameters (Å^2^)

**Table:**

	*U*^11^	*U*^22^	*U*^33^	*U*^12^	*U*^13^	*U*^23^
C1	0.0185 (8)	0.0204 (8)	0.0175 (8)	0.0011 (6)	0.0052 (6)	−0.0007 (6)
O1	0.0173 (6)	0.0346 (8)	0.0269 (7)	0.0001 (5)	0.0040 (5)	0.0061 (5)
O2	0.0227 (6)	0.0285 (7)	0.0251 (6)	0.0005 (5)	0.0053 (5)	0.0084 (5)
C2	0.0190 (8)	0.0200 (8)	0.0165 (8)	0.0030 (6)	0.0038 (6)	−0.0018 (6)
C3	0.0213 (8)	0.0251 (9)	0.0195 (8)	0.0016 (7)	0.0054 (6)	−0.0001 (6)
C4	0.0267 (9)	0.0253 (9)	0.0219 (8)	0.0031 (7)	0.0073 (7)	0.0034 (7)
C5	0.0246 (9)	0.0249 (9)	0.0175 (8)	0.0057 (7)	0.0029 (7)	−0.0022 (6)
C6	0.0175 (8)	0.0265 (9)	0.0237 (8)	0.0019 (7)	0.0032 (7)	−0.0034 (7)
C7	0.0196 (8)	0.0226 (9)	0.0211 (8)	0.0012 (7)	0.0052 (6)	−0.0003 (6)
C8	0.0238 (9)	0.0262 (9)	0.0234 (8)	0.0025 (7)	0.0045 (7)	0.0031 (7)
C9	0.0264 (9)	0.0248 (9)	0.0221 (8)	0.0011 (7)	0.0062 (7)	0.0004 (7)
C10	0.0220 (9)	0.0243 (9)	0.0187 (8)	0.0044 (7)	0.0028 (6)	−0.0041 (6)
C11	0.0216 (9)	0.0259 (10)	0.0265 (9)	−0.0002 (7)	0.0055 (7)	−0.0057 (7)
C12	0.0347 (10)	0.0217 (9)	0.0265 (9)	0.0025 (8)	0.0104 (8)	0.0006 (7)
C13	0.0280 (9)	0.0233 (9)	0.0215 (8)	0.0116 (7)	−0.0018 (7)	−0.0012 (7)
C14	0.0188 (8)	0.0273 (9)	0.0290 (9)	0.0019 (7)	0.0038 (7)	−0.0072 (7)
C15	0.0260 (9)	0.0229 (9)	0.0232 (8)	0.0013 (7)	0.0067 (7)	−0.0006 (7)
F1	0.0413 (7)	0.0402 (8)	0.0330 (7)	0.0202 (6)	−0.0063 (5)	0.0036 (5)

### Geometric parameters (Å, °)

**Table:**

C1—O1	1.252 (2)	C8—C9	1.330 (3)
C1—O2	1.291 (2)	C8—H8	0.9500
C1—C2	1.479 (2)	C9—C10	1.471 (2)
O2—H2	1.04 (4)	C9—H9	0.9500
C2—C3	1.392 (3)	C10—C15	1.392 (3)
C2—C7	1.393 (3)	C10—C11	1.402 (3)
C3—C4	1.382 (2)	C11—C12	1.390 (3)
C3—H3	0.9500	C11—H11	0.9500
C4—C5	1.394 (3)	C12—C13	1.380 (3)
C4—H4	0.9500	C12—H12	0.9500
C5—C6	1.401 (3)	C13—F1	1.363 (2)
C5—C8	1.473 (2)	C13—C14	1.369 (3)
C6—C7	1.389 (2)	C14—C15	1.389 (3)
C6—H6	0.9500	C14—H14	0.9500
C7—H7	0.9500	C15—H15	0.9500
			
O1—C1—O2	123.07 (15)	C9—C8—H8	116.9
O1—C1—C2	120.16 (15)	C5—C8—H8	116.9
O2—C1—C2	116.78 (15)	C8—C9—C10	125.99 (18)
C1—O2—H2	112 (2)	C8—C9—H9	117.0
C3—C2—C7	119.79 (15)	C10—C9—H9	117.0
C3—C2—C1	119.41 (16)	C15—C10—C11	118.25 (16)
C7—C2—C1	120.80 (16)	C15—C10—C9	118.07 (17)
C4—C3—C2	120.05 (17)	C11—C10—C9	123.68 (17)
C4—C3—H3	120.0	C12—C11—C10	120.96 (18)
C2—C3—H3	120.0	C12—C11—H11	119.5
C3—C4—C5	121.11 (18)	C10—C11—H11	119.5
C3—C4—H4	119.4	C13—C12—C11	118.08 (18)
C5—C4—H4	119.4	C13—C12—H12	121.0
C4—C5—C6	118.38 (16)	C11—C12—H12	121.0
C4—C5—C8	117.69 (17)	F1—C13—C14	118.85 (18)
C6—C5—C8	123.92 (17)	F1—C13—C12	118.02 (18)
C7—C6—C5	120.88 (17)	C14—C13—C12	123.12 (17)
C7—C6—H6	119.6	C13—C14—C15	118.00 (17)
C5—C6—H6	119.6	C13—C14—H14	121.0
C6—C7—C2	119.77 (17)	C15—C14—H14	121.0
C6—C7—H7	120.1	C14—C15—C10	121.58 (18)
C2—C7—H7	120.1	C14—C15—H15	119.2
C9—C8—C5	126.22 (18)	C10—C15—H15	119.2
			
O1—C1—C2—C3	−6.5 (2)	C6—C5—C8—C9	−7.2 (3)
O2—C1—C2—C3	173.26 (15)	C5—C8—C9—C10	−179.93 (17)
O1—C1—C2—C7	174.23 (16)	C8—C9—C10—C15	−175.06 (18)
O2—C1—C2—C7	−6.0 (2)	C8—C9—C10—C11	5.5 (3)
C7—C2—C3—C4	−0.1 (3)	C15—C10—C11—C12	−0.1 (3)
C1—C2—C3—C4	−179.31 (16)	C9—C10—C11—C12	179.26 (17)
C2—C3—C4—C5	−1.0 (3)	C10—C11—C12—C13	0.4 (3)
C3—C4—C5—C6	1.1 (3)	C11—C12—C13—F1	−179.25 (16)
C3—C4—C5—C8	−179.83 (16)	C11—C12—C13—C14	0.1 (3)
C4—C5—C6—C7	−0.2 (3)	F1—C13—C14—C15	178.64 (16)
C8—C5—C6—C7	−179.19 (17)	C12—C13—C14—C15	−0.7 (3)
C5—C6—C7—C2	−0.9 (3)	C13—C14—C15—C10	0.9 (3)
C3—C2—C7—C6	1.0 (3)	C11—C10—C15—C14	−0.5 (3)
C1—C2—C7—C6	−179.80 (15)	C9—C10—C15—C14	−179.93 (16)
C4—C5—C8—C9	173.72 (19)		

### Hydrogen-bond geometry (Å, °)

**Table:**

*D*—H···*A*	*D*—H	H···*A*	*D*···*A*	*D*—H···*A*
O2—H2···O1^i^	1.04 (4)	1.57 (4)	2.610 (2)	174 (3)

Symmetry codes: (i) −*x*+2, −*y*, −*z*+2.
